# Theory and Practice of Medicine

**Published:** 1877-06

**Authors:** 


*Theory and Practice of Medicine.* By JOHN SYER BRISTOWE, M. D., Lonel, F. R. C. P. Physician to St. Thomas Hospital, Joint Lecturer at the School, and examiner in Medicine to the Royal College of Surgeons, formerly examiner in Medicine to the University of London, and lecturer on general pathology, and on physiology at St. Thomas Hospital. Edited with notes by JAMES H. HUTCHINSON, M. D., one of the attending physicians to the Pennsylvania Hospital, physician to the Children's Hospital, Philadelphia. etc. Philadelphia: Henry C. Lea, 1876,

This book is offered to medical students as a text book, and to the profession. From what we have been able to read of it, we think it adapted to the medical students in very high degree, while the experienced practitioner will take great pleasure and derive much benefit by careful perusal. The plain and practical descriptions of disease are so attractive to those having had some personal knowledge of origin, progress and termination of diseased processes, that too much cannot be said in favor of the work. It shows that the author speaks of conditions he has himself seen, studied and treated. His descriptions are "true to life." His pen pictures of diseased conditions, are beautiful, faithful, complete and instructive. They compose the attractions of the book in great degree. A wide range of subjects is considered, and the student or physician will here find full description of nearly all known disease, with mention of the various medicines used in its cure, without space to particularise, we can say in few words that the work is eminently adapted to the wants of both student and practitioner, As a book for the medical student, it is unsurpassed. As a standard work upon theory and practice of medicines, it is comprehensive, condensed, practical, and in all respects, "first rate."

